# Environment‐driven changes in diversity of riparian plant communities along a mountain river

**DOI:** 10.1002/ece3.7472

**Published:** 2021-03-25

**Authors:** Nihaib Flores‐Galicia, Irma Trejo, Neptalí Ramírez‐Marcial

**Affiliations:** ^1^ Posgrado en Ciencias Biológicas Universidad Nacional Autónoma de México Unidad de Posgrado Circuito de Posgrados Ciudad Universitaria Ciudad de México México; ^2^ Instituto de Geografía Circuito de la Investigación Científica Ciudad Universitaria Universidad Nacional Autónoma de México Ciudad de México México; ^3^ Departamento de Conservación de la Biodiversidad El Colegio de la Frontera Sur San Cristóbal de Las Casas Chiapas México

**Keywords:** altitudinal gradient, diversity of species, longitudinal dimension, mountain river, River, River Collector Hypothesis

## Abstract

The study of changes in species richness and composition along rivers has focused on large spatial scales. It has been ignored that in different sections of the river (high mountain area, middle zone, and mouth of the river) the specific environmental conditions can generate different longitudinal patterns of the species richness and composition. In this study, we determine whether species richness and composition of the riparian plant communities change along a mountain river and whether these changes are related to environmental variables. We expect an increase in species richness and turnover along the river, that the upstream communities would be a subset of the downstream communities, and that such would be related to edaphic and hydrologic conditions. To test this, we sampled three strata of the riparian vegetation (upper: individuals with <1 cm of ND, middle: individuals with >1 cm of ND, low: individuals with >1 m tall) in a set of 15 sites that we place along a mountain river. Additionally, we recorded topographic, hydrological, morphological, and soil variables. We performed correlation analyzes to determine whether changes in species richness and turnover were related to increased distance to the origin of the river. Also, we obtained the nestedness and evaluated the importance of environmental variables with GLM, LASSO regression, and CCA. With the increase in distance, the species richness decreases in the upper stratum, but not in the middle and the low stratum (although the highest values were observed near the origin of the river), the turnover increase in all strata and the upstream communities were not a subset of the downstream communities. The changes in species richness and composition were related to topographic (altitude), hydrological (flow), and edaphic (conductivity and pH) variables. Our results indicate that at small spatial scales the patterns of richness and composition differ from what has been found at larger spatial scales and that these patterns are associated with environmental changes in the strong altitude gradients of mountain rivers.

## Graphical abstract

Along the rivers, it is possible to observe patterns in the diversity of plants. We find that in mountain rivers the species richness decreases; there is an increase in the turnover and absence of nesting of the communities along the rivers. These patterns of diversity are determined by altitudinal contrasts and changes in hydrological and edaphological characteristics that contrast with what has been found in previous works.

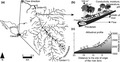

## 1 INTRODUCTION

Riparian plant communities are considered of central importance for conservation since they are one of the most diverse communities in the world (Mouw & Alaback, 2003; Naiman & Decamps, 1997; Sabo et al., 2005; Tab acchi et al., 1996). Several studies have found patterns in the richness and composition of riparian plant communities associated with distance gradients with respect to the origin of the river or the mouth of the river (Warfe et al., 2013). In general, these studies have observed that species richness increases downstream to a maximum point in the middle zone and then decreases toward the mouth of the river (Honnay et al., 2010; Nilsson et al., 1994; Tab acchi et al., 1996). As to the composition of the communities of riparian plants, differences have been observed between the composition of the communities of the origin of the river and the composition of the communities of the mouths (turnover), as well as nestedness of the composition of species, being the composition of upstream communities a subset of the composition of downstream communities (Honnay et al., 2010; Kuglerova et al., 2015).

The changes in species richness and composition of riparian plant communities along the rivers are the results of the effect that different processes and environmental conditions have on the species of these communities (Kuglerova et al., 2015; Myers & Harms, 2009; Naiman et al., 2010; Tonkin et al., 2018; Warfe et al., 2013). The most important environmental conditions for riparian plant communities are the soil conditions (conductivity and moisture) and hydrological conditions (flow rate and variations in the water flows) (Kuglerova et al., 2015; Myers & Harms, 2009), while, among the processes, the propagules dispersal stands out (Kuglerova et al., 2015; Myers & Harms, 2009; Nilsson et al., 2010). Along the rivers, directional changes of these variables and processes are closely related to the increase in the distance to the origin of the river (Warfe et al., 2013). For example, with increasing distance to the origin of the river, the amount of water moving through the rivers increases, which in turn also increases the transport capacity of sediments and propagules through the river (Kuglerova et al., 2015).

The "River Collector Hypothesis" (RCH) summarizes the importance of environmental conditions and the process of propagule dispersal in the changes of species richness and composition of riparian plant communities that are observed along the rivers, from the site of origin of the river to mouth of the river (Nilsson et al., 2010). The RCH assumes that (a) the number of species and propagules transported by the river and deposited in the riparian zones increase proportionally with the distance to the origin of the river, (b) the environmental conditions vary along rivers, and (c) there is a gradient in the intensity and magnitude of the disturbance generated by the increase in floods downstream, which restricts the establishment of the species toward the mouth of the river but facilitates its establishment in the areas near the site of origin of the river by creating spaces for the establishment of dispersed propagules (Nilsson et al., 1994, 2010).

Recent studies indicate that during the first phases of community assembly, the propagule dispersal determines the species richness patterns of riparian communities, being the areas with the highest deposition of propagules where species richness is higher (Fraaije et al., 2017). The effect of the propagule dispersal on diversity is overcome by environmental filters that affect the survival of individuals in later phases of community assembly (Fraaije, ter Braak, Verduyn, Breeman, et al., 2015; Fraaije et al., 2015b). This differential effect at different phases of community assembly could be reflected in variations in the patterns of species richness and composition between the plant strata of the riparian communities along the rivers.

These studies and the RCH have evaluated changes in species richness and composition patterns on large spatial scales. For example, Warfe et al. (2013) worked considering rivers with a length of more than 300 km, while Tab acchi et al. (1996) considered rivers of 100–600 km. On the other hand, the studies that have been carried out at small spatial scales also consider small altitude differences between the site of origin of the river and its lowest point. For example, Kuglerova et al. (2015) evaluated riparian communities in a river ~20 km long and with an altitudinal gradient that ranged from 114–405 m a. s. l. It is unknown whether specific edaphic, geomorphological, hydrological, and topographic conditions in the different sections of a river can generate differential patterns in diversity that do not agree with what has been found on large spatial scales or with low altitudinal gradients. Considering this, the study of rivers associated with mountain systems is important, since they have unique characteristics that are given by the wide confinement sections and the steep slopes, which increase their capacity to transport material downstream, while reducing the capacity of material deposition toward the riparian zones (Jacobsen, 2008; Meyer et al., 2007; Wohl, 2010). These characteristics of mountain rivers can reduce their retaining capacity of propagules in the riparian zones and with this decrease the nestedness of the riparian plant communities; on the other hand, greater energy in mountain rivers could have a negative effect on the species richness by increasing the intensity of the disturbance.

In the present study, we tested whether there are changes in the species richness and composition of riparian plant communities along the upper part of a mountain river in southern Mexico. To do so, we characterized the riparian plant communities considering three vegetation strata (upper: individuals with <1 cm of ND, middle: individuals with >1 cm of ND, low: individuals with >1 m tall) in sites that were in a distance gradient with respect to the origin of the river. We expected that (a) species richness increases with distance to the origin of the river; that there are changes in the composition, (b) specifically that the turnover of species increases with distance from the origin of river, and (c) that upstream community composition constitutes a subset of the species found in downstream communities; finally, we expected that (d) changes in species richness and composition will be associated with changes in edaphic and hydrologic conditions in the riparian zones.

## 2 METHODS

### 2.1 Study area

**FIGURE 1:**
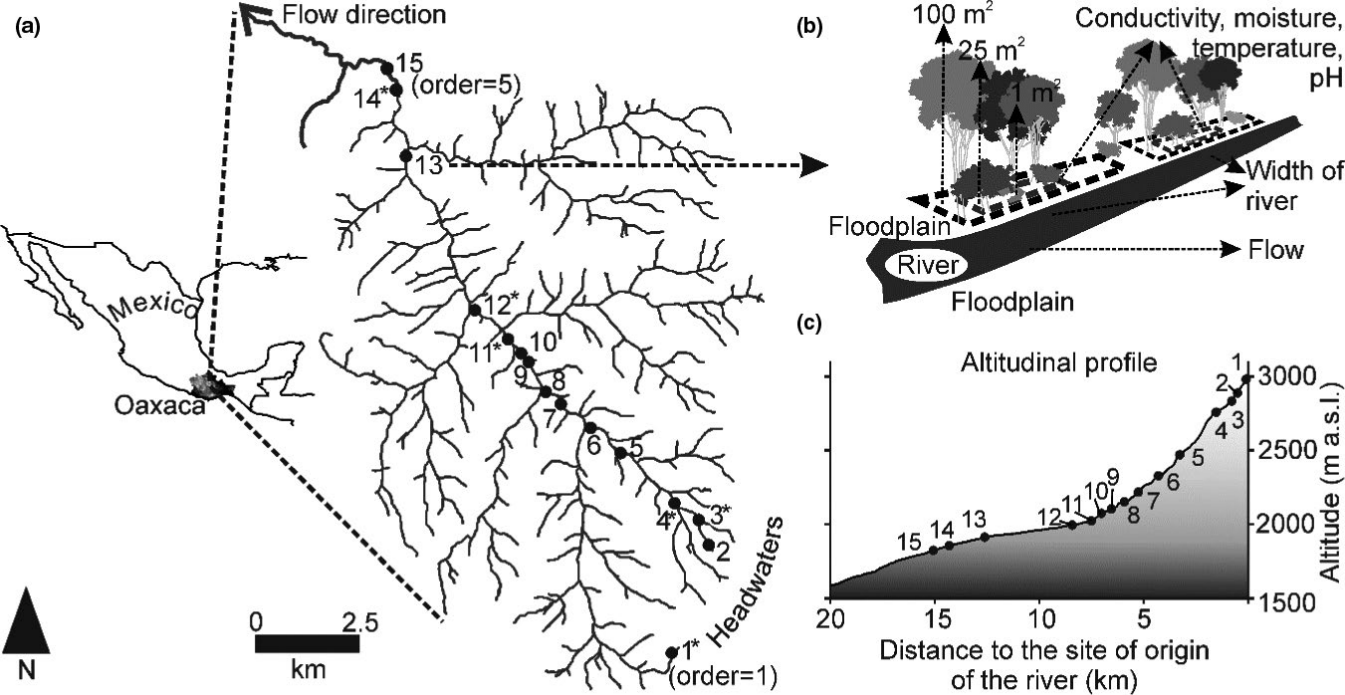
Location of the sampling sites along a river in the upper part of the Papaloapan River basin, Oaxaca, Mexico. (a) 15 sites were established along one river, and the Strahler stream order is shown in parentheses for site 1 and 15. (b) In each site, riparian vegetation was inventoried in two sets of plots of different sizes. The upper stratum in plots of 100 m^2^, the middle stratum in plots of 50 m^2^, and the low stratum in plots of 1 m^2^. (c) The position of the sites in an altimetric profile is shown. *Sites in which all the environmental variables were recorded

The study was conducted in a river of the upper part of the Papaloapan River basin in the state of Oaxaca, Mexico. In its first 16 km, the river drains from a maximum altitude of 3,216 m a. s. l. toward a minimum altitude of 1,833 m a. s. l. The stream order (following Strahler, 1957) goes from the first order in the upper zone to the fifth order in the lowest zone (Figure 1). The climate in the upper zone corresponds to humid temperate with summer rains and that in middle and low zones corresponds to subhumid temperate with summer rains. The type of soil of the upper and middle zones corresponds to Cambisol and Regosol, respectively, where the lower part has Fluvisol, Cambisol, and Regosol (INEGI, 2004). The upland forest communities are *Abies hickelii* forests and *Pinus hartwegii* forests in the highest zone and mixed forests of *A. hickelli*‐*Pinus patula* and of *Pinus pseudostrobus* var. *apulcensis*‐*Quercus crassifolia* toward the zones medium and low (Piña & Trejo, 2014).

### 2.2 Sampling sites on a distance gradient

In the river, 15 sampling sites were established in the first 16 km of the stream (following the path of the river) (Figure 1a). In order to determine the relationship between the distance to the origin of the river with changes in richness and composition (turnover and nestedness), their position was calculated by determining the distance from each sampling site to the river source site.

### 2.3 Vegetation survey

Fieldwork was carried out during the two seasons with the greatest floristic contrast in the area (dry season = March 2017 and rainy season = November 2017). To floristically characterize the riparian communities that develop along the river, at each sampling site all vascular plant species were recorded in plots of different sizes depending on the stratum studied; in each parcel, all individuals per species (except for low stratum) and his normalized diameter (ND = diameter at height of 1.3 m) were registered. Due to the rugged topography of the area, the plots were placed on a riverbank. The strata were separated because different life forms dominated the upper and middle layers (dominated by trees and shrubs) and the lower layer (dominated by herbaceous plants). For the upper stratum, all individuals with more than 1 cm of ND were measured in two plots of ~ 100 m^2^ (3x33.3 m). For the middle stratum, all the individuals smaller than 1 cm of ND were measured in two plots of 50 m^2^ (2x25 m). Finally, for the lower stratum, the coverage of all individuals <1 m tall were recorded in two plots of 1 m^2^ (1 × 1 m).

### 2.4 Environmental variables

We selected a set of sites covering the distance gradient to the river origin (sites 1, 2, 3, 10, 11, 14) as well as the prevailing climatic and soil conditions in the area to analyze whether changes in species richness and composition were related to changes in environmental variables. At each of these sites, we recorded altitude as well as morphological, hydrological, and edaphological variables. For recording environmental variables, the sites were visited on four occasions between March and November 2017, except for flow, which was only recorded in the rainy season (November 2017). For the morphological variables, we obtained the average channel width at each site by measuring the distance between the growth lines of the vegetation on both banks of the river at three sites.

Regarding the hydrological variable, was calculated the river flow at each of the sample sites using the salt hit method. This method consists of the use of a chemical marker (NaCl) that allows the recording of changes in electrical conductivity (EC) in the water from the river through time. With these measurements, we subsequently calculate the flow using the formula (Moore, 2005): *Q* = *V*/*k*Δ*t*∑_n_[EC(*t*)–EC*_bg_*]. Where *Q* is flow, *k* is the calibration constant, *t* is any time during the registration of the chemical marker, EC(*t*) is the electrical conductivity measured in time *t,* and EC*_bg_* is the initial electrical conductivity of the river. In the field, all EC measurements were taken at a constant distance at all sites (20 m to the dumping site). The volume of NaCl varies depending on the size of the river (site 1 = 0.5 kg, site 14 = 5 kg). The length of time the EC measurements lasted depended on the speed at which the entire volume of the chemical marker discharged into the river passed the measurement point at each site (site 1 = 395 s, site 14 = 145 s). These measurements of the river flow were made once in the rainy season.

For the edaphic characterization of the riparian zone, soil measurements of electrical conductivity, moisture content, pH, and temperature were recorded with a multiparameter and an electronic sensor throughout one year (Figure 1b). For the edaphic variables, it was evaluated whether there were differences between seasons (dry and rainy seasons) with a Mann–Whitney test (Appendix S1). For those variables in which differences were observed between seasons, its relationship with the distance to the origin of the river was evaluated with a Pearson correlation analysis. Prior to the correlation analysis and due to differences in the measurement scales of the edaphic parameters, the variables were transformed to a logarithmic scale (Appendix S2).

### 2.5 Statistical analysis

All the analysis was made for each stratum with the help of the R ver. 3.5.2 software (R Core Team 2018).

For species richness, the R‐package iNext was used to perform rarefaction analysis and calculated the Hill number of order 0 (*q*0, species richness). Rarefaction allows the direct comparison of diversity values between differently sampled sites by calculating an expected number of species based on fixed sample size (Hsieh et al., 2016). With the species richness values obtained by rarefaction (*q*0), a Spearman correlation analysis was performed to determine whether the changes in species richness were related to the increase in the distance to the origin site of the river.

Changes in the composition of species along the river were analyzed by calculating two complementary measures of *β* diversity (Kuglerova et al., 2015). The first measure was the turnover; it is a measure of the change in the structure of one site to another considering a spatial, temporal, or environmental gradient (Anderson et al., 2011; Nekola & White, 1999). To measure the turnover in the distance gradient to the origin of the river, we performed a distance decay analysis using a matrix with the species presence per site to obtain Simpson's dissimilarity coefficient (*β*_sim_) as a measure of replacement and a matrix with transformed environmental variables (log) to obtain the Euclidean distance between sites as a measure of environmental dissimilarity in the distance gradient. Simpson's dissimilarity coefficient was used because this index accounts only for turnover between communities (Simpson, 1943). We determined the correlation between Simpson's dissimilarity coefficient with the increase in the distance between sites and with the environmental dissimilarity through a Spearman correlation analysis. Spearman correlation analysis was performed to assess whether environmental dissimilarity increases with distance between sites.

The second measure was the nestedness, which occurs when the species of a site are a subset of the species present in another site (Almeida‐Neto et al., 2008). To calculate the nestedness, the NODF (nestedness metric based on overlapping and decreasing fill) was obtained following the formula: NODF = ∑N_paired_/[*n*(*n*–1)/2]+[*m*(*m*–1)/2]. Where *N*_paired_ is a degree of paired nestednesss, *n*(*n*–1)/2 and *m*(*m*–1)/2 is the degree of nestedness for n columns (species) and m (sites) rows, respectively. The NODF takes values ranging from 0 to 100, a value of 100 indicates total nestedness of the communities (Almeida‐Neto et al., 2008). The values of NODF were obtained using the R‐package vegan (Oksanen et al., 2013). Site 1 was excluded from the nestedness analysis because it is in a different stream.

The species relative importance value (RIV) was obtained for each site. The RIV is a comprehensive indicator that used to determine the overall importance of each species in the community structure (Curtis & McIntosh, 1951; Ding et al., 2018; Méndez‐Toribio et al., 2014). Because for the low stratum a measure of abundance was not recorded at each site, the RIV calculation was different for the low stratum; the calculation for each stratum is described below. In the upper and middle strata, it was obtained with the formula RIV= ∑RF_i_+∑RA_i_+∑RD_i_/3. Where RF_i_ is the relative frequency of species *i*, RA_i_ is the relative abundance of species *i*, and RD_i_ is the relative dominance of species *i* (obtained with the basal area). For the low stratum, the RIV was obtained with: RIV= ∑RF_i_+∑RD_i_/2.

Data from six sampling sites covering the distance gradient (sites 1, 2, 3, 10, 11, 14) were used to evaluate the relationship between species richness and composition of plant communities with environmental variables. To avoid collinearity between the environmental variables, a Pearson correlation analysis was performed with the variables transformed on a logarithmic scale (Appendix S2). Three edaphic variables (moisture and conductivity of the rainy season and pH), flow, and altitude were selected to elaborate the generalized linear models (GLM) and the canonical correspondence analysis (CCA).

We used GLMs (with a negative binomial distribution) to test whether there was a relationship between environmental variables and species richness. The GLMs were made with R‐package lme4 (Bates et al., 2014). The best models were selected considering the maximum likelihood criterion using the corrected Akaike's information criterion (AICc) and the deviance explained by the model. When the difference of AICc between models was <2, both models were considered to be equally supported, and in these cases, the model with the greatest deviance explained was chosen. Furthermore, when the best model selected through the GLMs was a model with the interaction, a hierarchical partitioning was performed to determine the proportion of variance independently explained by each variable in the model using the R‐package hier.part (Nally & Walsh, 2004). Even after the correlation analysis between the variables, some of the selected variables for the GLMs were found to have a strong correlation. The use of a technique less sensitive to the effect of collinearity, the least absolute shrinkage and selection operator (LASSO) (Dormann et al., 2013), will allow us to contrast the results obtained with the GLMs and graphically evaluate the effect of the collinearity (Appendix S3). The LASSO is a penalized regression technique that constrains the size of estimated coefficients (shrink the effect of less important predictor variables to zero) by minimizing the deviance of the linear model fit (Friedman et al., 2010). The parameter controlling the extent of shrinkage (λ) was determined using a cross‐validation. The LASSO regression was conducted using the R‐package glmnet (Friedman et al., 2010).

To evaluate the influence of environmental variables on the composition of species of riparian plant communities, we conducted a Canonical Correspondence Analysis (CCA). The CCA is an ordination technique that evaluates the associations between a set of environmental variables and the response variable, in this case, data on the composition of communities (Ter Braak, 1987). For the CCA analysis, we used a matrix with the data of the species (abundance of the species in the case of the upper and middle strata and percentage of coverage by species for the low stratum) and a matrix with the values of the environmental variables transformed to a logarithmic scale (log10). CCAs were made using the R‐package vegan (Oksanen et al., 2013).

## 3 RESULTS

### 3.1 Species richness

**FIGURE 2:**
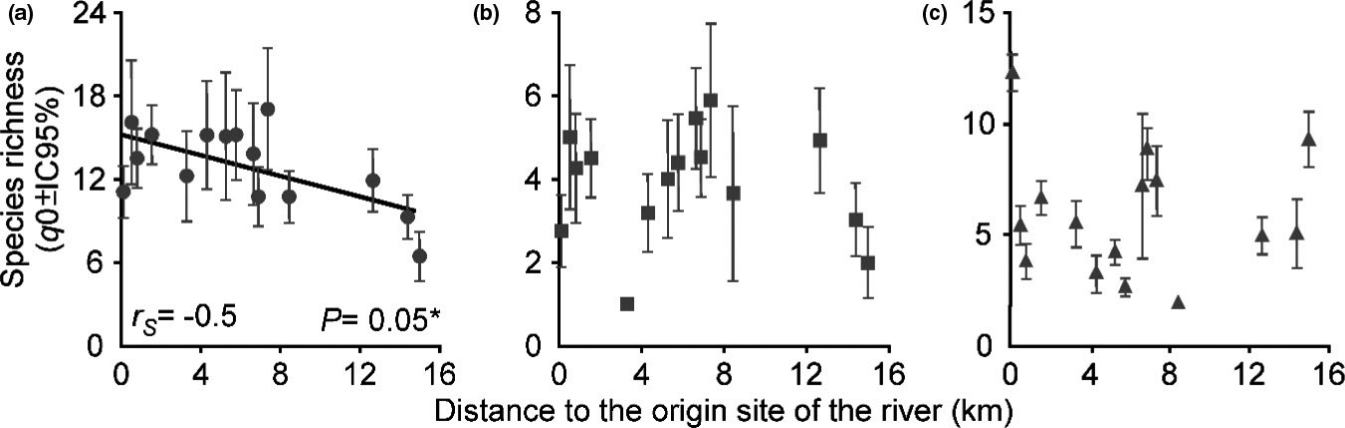
Relationship between species richness of riparian plant communities and geographical distance from the origin of the river. The species richness (*q*0) in the upper stratum decreases with distance to the origin of the river, for the low stratum the richness in site 1 was higher than the rest of the sites. (a) Upper stratum. (b) Middle stratum. (c) Low stratum. (*) Significant correlations

In total, 150 species of vascular plants belonging to 63 families were registered (upper stratum: 71 species of 36 families; middle stratum: 44 species of 25 families; low stratum: 98 species of 44 families). We detected a negative and significant correlation between the species richness and the distance to the origin of the river for the upper stratum (*r_S_* = −0.5, *p* = 0.05). For the middle stratum, we did not observe a significant relationship between the species richness and the position of the sites in the river (*r_S_* = 0.01, *p* = 0.96). For the low stratum, we also do not observe a significant relationship between the species richness and the position of the sites in the river (*r_S_* = 0.01, *p* = 0.96) (Figure 2).

### 3.2 Community composition

With the increase in environmental dissimilarity, the turnover of species in the upper stratum increases (*r_S_* = 0.95, *p *= < 0.001) but not in the middle (*r_S_* = 0.40, *p* = 0.37) and in the low stratum (*r_S_* = 0.14, *p* = 0.61). The results show that with the increase in the geographical distance between sites, the differences in environmental conditions increase (*r_S_* = 0.82, *p *= < 0.001).

**FIGURE 3:**
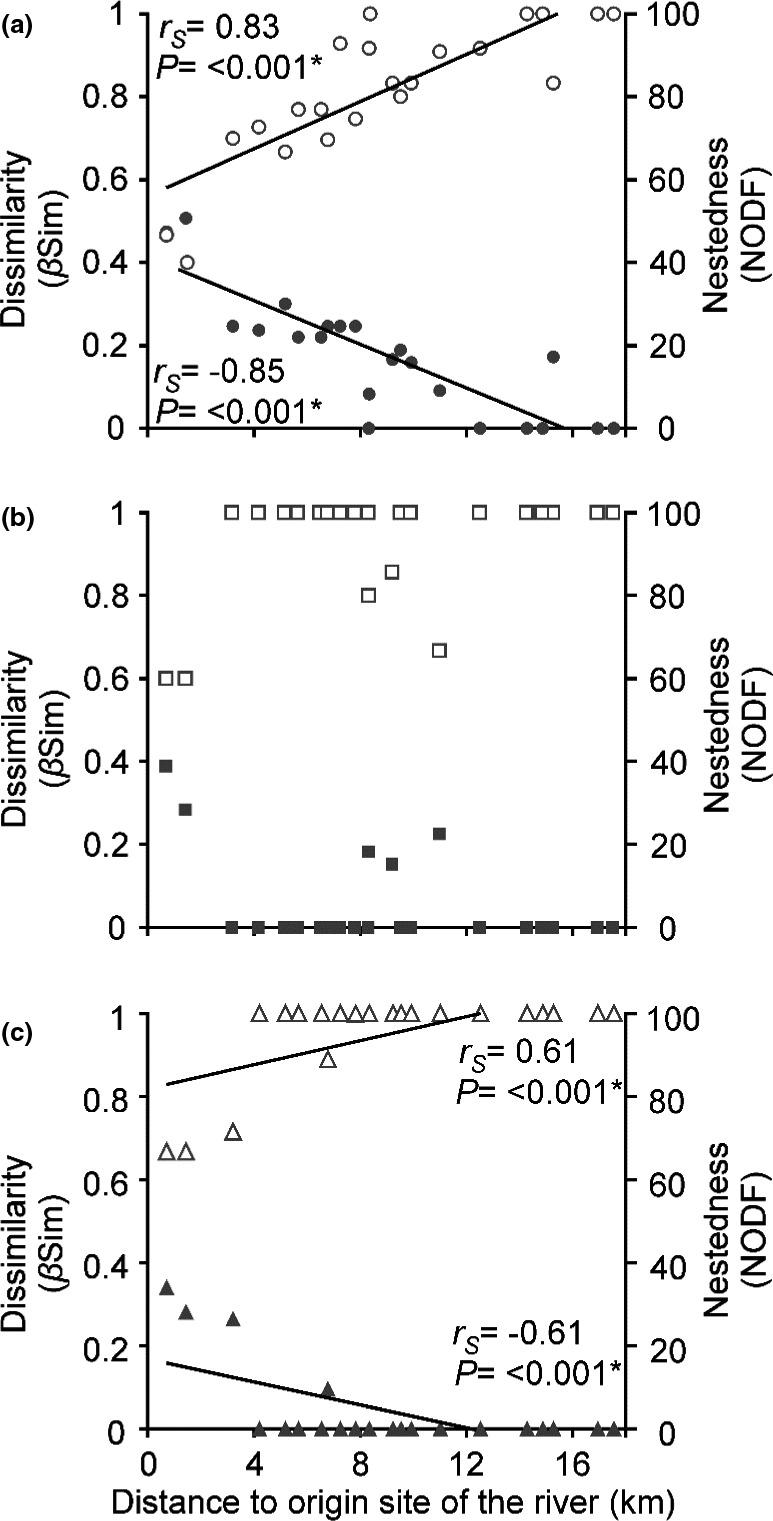
Relationship between community dissimilarity, nestedness, and distance to the origin of the river. Dissimilarity increases and nestedness decreases with the distance to the origin of the river in upper stratum (a) and low stratum (c) but not for the middle stratum (b). The filled circles, squares, and triangles are the dissimilarity values while the unfilled ones are the nestedness values. (*) Significant correlations

We observed that with the increase in the geographic distance to the sites near to the origin of the river there is an increase in the turnover of species for the upper stratum (*r_S_* = 0.83, *p *= < 0.001) and for the low stratum (*r_S_* = 0.61, *p *= < 0.001) but not in the middle stratum (*r_S_* = 0.21, *p* =.28). The lowest turnover values for all strata were observed between the closest sites (site 2, 3, 4) (Figure 3). Regarding nestedness, we observe that it decreased with the increasing distance to the sites near to the origin of the river for the upper stratum (*r_S _*= −0.83, *p *= < 0.001) and for the low stratum (*r_S _*= −0.61, *p *= < 0.001) but not in the middle stratum (*r_S _*= −0.21, *p* = 0.28). The highest nestedness values for all strata were observed between the closest sites (site 2, 3, 4). (Figure 3).

**FIGURE 4:**
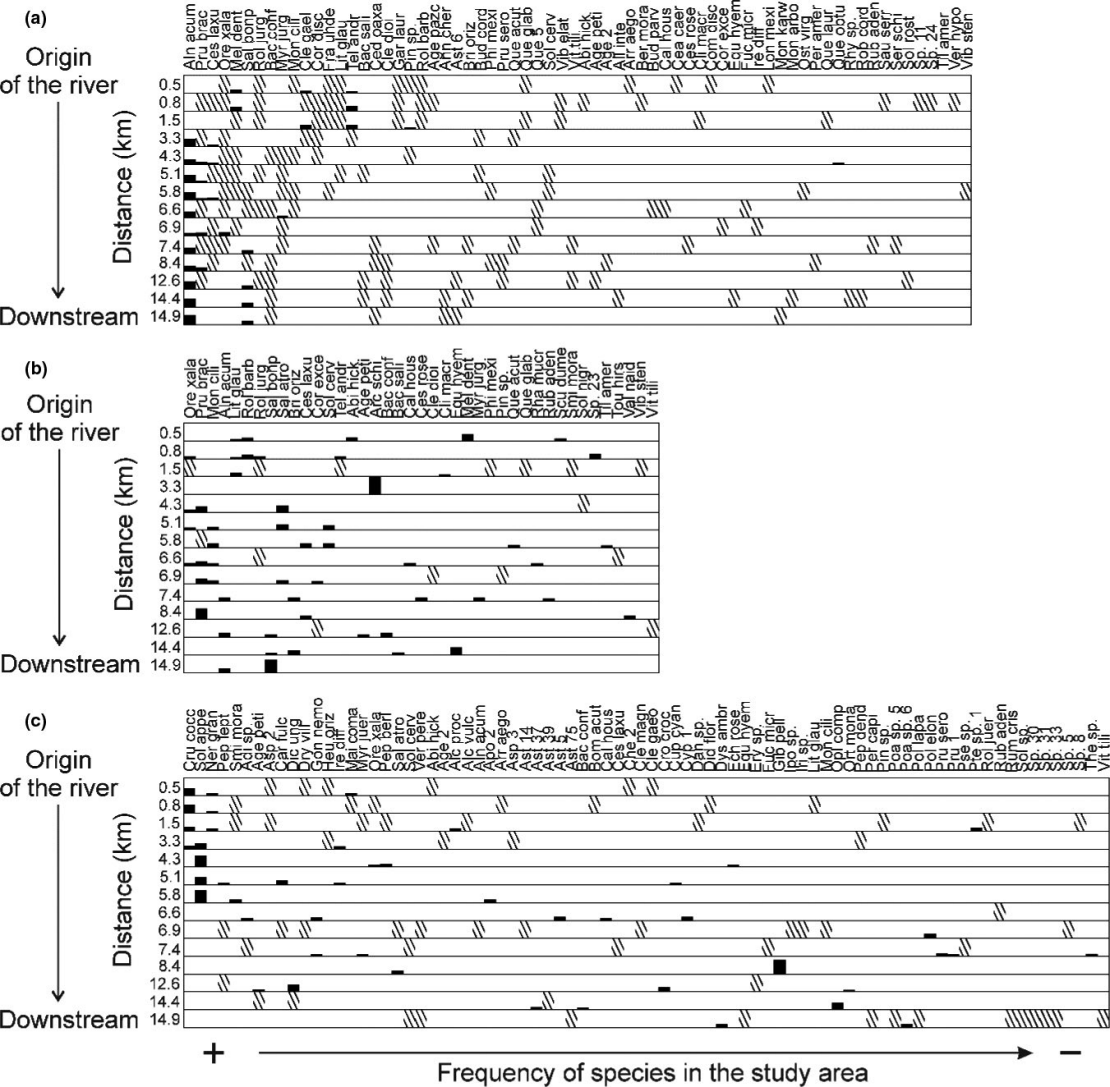
Presence of species and RIV in the sampling sites from the origin of the river to downstream. No nestedness of the species composition of the sites near the river starting point is observed in the composition of the downstream sites. (a) Upper stratum, (b) middle stratum, (c) low stratum. The species are arranged in the order of frequency, the most frequent species on the left side and the less frequent species on the right side. The RIV is presented for species with RIV > 10 (black bar, the size of the bar represents its VIR value), species with RIV < 10 as shown with diagonal lines. The names of the species are shown in Appendix S4

There is no nestedness in the composition of the river source sites into the downstream sites (upper stratum NODF = 22.59, middle stratum NODF = 12.22 low stratum NODF = 7.47) (Figure 4). The results of the analysis of nestedness and species turnover indicate that no species is distributed along the entire river. Species that are distributed in lowland areas are not found in areas close to the origin of the river such as *Alnus acuminata*, *Salix bonplandiana,* or *Baccharis conferta* and species from areas near to the origin of the river such as *Oreopanax xalapensis* and *Meliosma dentata* do not reach the lowland areas of the river. Therefore, the species that are part of the communities near the origin of the river and the communities of downstream river are different (Figure 4).

The species from the upper stratum that were observed only in one site had a RIV < 10, in contrast in the middle and lower stratum, several species present only in one site had a RIV > 10. The most frequent species of the upper, middle, and lower strata (such as *Alnus acumiata*, *Oreopanax, xalapensis*, *Crusea coccinea,* and *Solanum appendiculatum*) had the highest RIV values in the sites where they were observed (Figure 4, Appendix S4).

### 3.3 Influence of environmental variables

Regarding environmental variables, the GLMs indicated that species richness in the upper stratum was not related to any of the environmental variables (EC was marginally significant). For this stratum, in the LASSO regression three variables were maintained, the most important being conductivity. In the middle stratum, both the GLMs and the Lasso regression indicate that was significantly positively related to pH. In the low stratum, the species richness was significantly negatively related to the interaction of altitude with flow, in this stratum hierarchical partitioning identified flow as the most important variable (55.42% of the variance explained), a similar result was obtained with the LASSO regression (Tables 1 and 2).

**TABLE 1:** Models describing the relation between the changes in species richness in the upper, middle, and low strata along the river with the environmental variables and hierarchical partitioning of environmental variables of models with interactions

Model	Predictors of model	*β_0_*	*β_1_*	*p*	AICc	*D* ^2^	*I*	Z
Upper stratum								
Rich ~ Alt : Con		−55.02	22.40	0.06	36.11	0.61		
	Alt						13.86	−0.44
	Con						86.14	1.75*
Rich ~ Con		−175.50	22	0.07	36.12	0.60		
Middle stratum								
Rich ~ pH		188.76	−156.14	0.04*	31.88	0.70		
Rich ~ Alt : Mc		−6.94	31.31	0.13	34.45	0.47		
	Alt						9.13	−0.74
	Mc						90.87	0.90
Low stratum								
Rich ~ Alt : Flo		−4.68	21.27	0.05	38.91	0.66		
	Alt						44.57	0.74
	Flo						55.43	1.00
Rich ~ Flo		−14.92	20.77	0.05	39.08	0.64		

Note:

*I* values represent the percentage of variance accounted for each predictor in a hierarchical partitioning model with *Z* scores indicating statistical significance. Models with the lowest AICc values are shown.

Abbreviations: Alt, altitude; Con, conductivity; Flo, flow; Mc, moisture content.

*Statistical significance.

**TABLE 2:** Coefficients of the regression between the environmental variables and the richness of species selected with the LASSO regression

	Upper	Middle	Low
*λ*	0.36	0.08	1.7
Intercept	3.1	−13.31	2.05
Alt	–	–	0.27
Con	−9.51	–	–
Flo	−0.01	–	−0.98
Mc	0.01		–
Ph	–	17.63	–

Note

Abreviations: λ, penalty parameter value, Alt, altitude; Con, conductivity; Flo, flow; Mc, moisture content.

**FIGURE 5:**
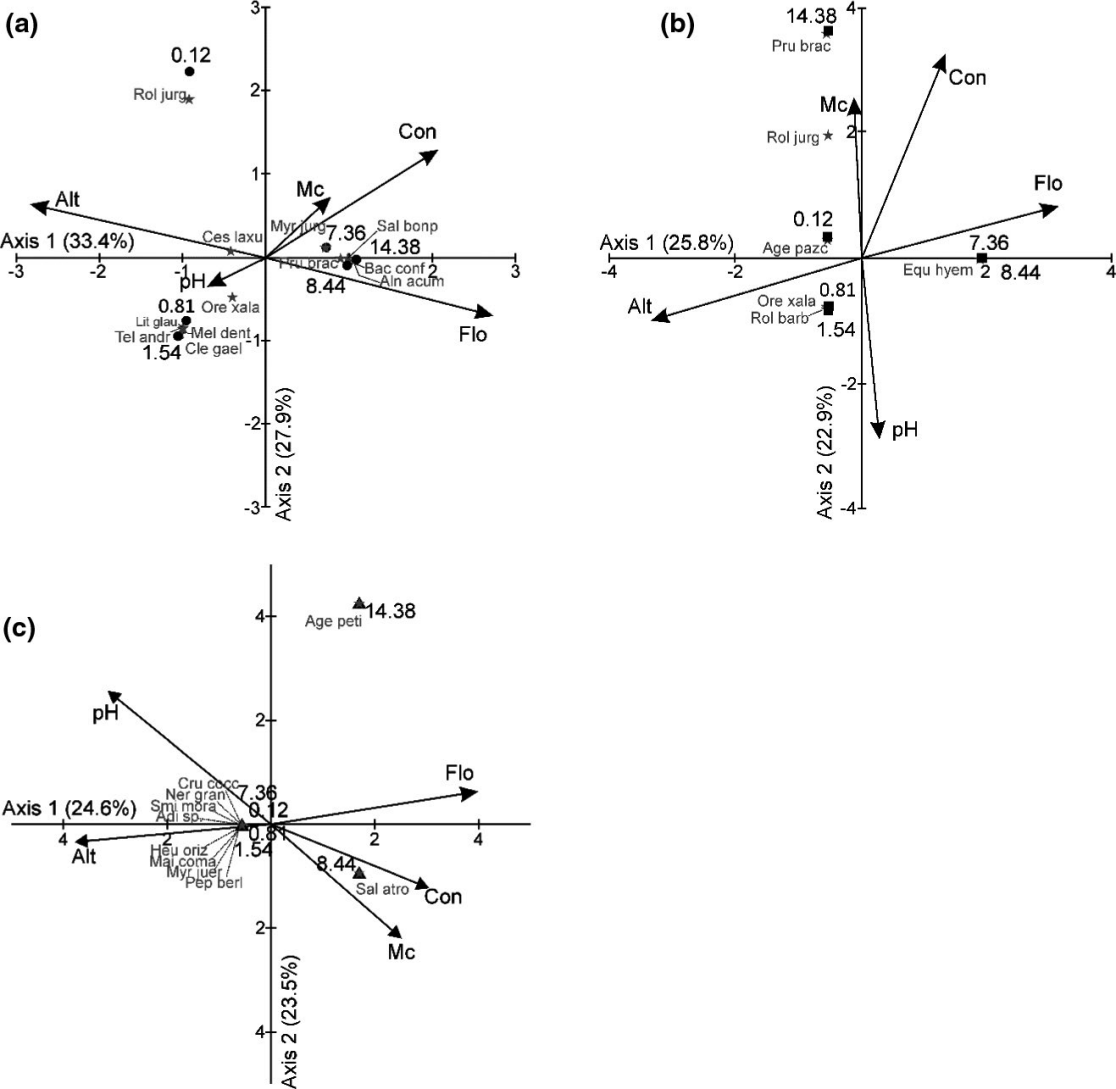
Ordination of the study sites based on the Canonical Correspondence Analysis, using the composition of species of sampling sites and environmental variables. The differences in the species composition of the sites near to the origin of the river and the downstream sites were related in the upper and in the middle stratum with flow, altitude, and conductivity; in the low stratum, it was related with flow and pH. (a) Upper–middle stratum. (b) Middle stratum. (c) Low stratum. Alt, altitude; Con, conductivity; Flo, flow; Mc, moisture content. The most frequent species in the study area are displayed (species names are in Appendix S4)

Finally, the CCA indicates that the first two axes explained 61.3% of the variation of the floristic composition of the upper stratum along the river (Figure 5 a). In axis 1 (explained variance = 33.4%), it was positively correlated with the flow (*r* = 0.93) and negatively with the altitude (*r*= −0.97). In axis 2 (explained variance = 27.9%), it was correlated positively with the conductivity (*r* = 0.43). For the middle stratum, the CCA indicated that the first two axes of ordination explained 48.7% of the variation of the floristic composition that we observe along the river (Figure 5 b). In axis 1 (explained variance = 25.8%), it was positively correlated with the flow (*r* = 0.79) and negatively with the altitude (*r*= −0.84). In axis 2 (explained variance = 22.9%), it was positively correlated with the conductivity (*r* = 0.82) and negatively correlated with pH (*r* = 0.73). For the low stratum, the CCA indicated that the first two axes of ordination explained 48.1% of the variation of the floristic composition that we observe along the river (Figure 5 b). In axis 1 (explained variance = 24.6%), it was positively correlated with the flow (*r* = 0.89) and negatively with the altitude (*r* = 0.76). In axis 2 (explained variance = 23.5%), it was positively correlated with pH (*r* = 0.57).

## 4 DISCUSSION

Different from our expectation, we found a negative relationship between species richness and distance to the origin of the river as well as that the nestedness component was not predominant; as such, we found no support for the "River Collector Hypothesis" (RCH) (Nilsson et al., 2010). Previous studies and the RCH hypothesized that the propagule dispersal process is central to explain the increase in species richness toward the intermediate zone of the rivers and the nestedness of the composition of upstream sites in the species composition of downstream sites (Kuglerova et al., 2015; Nilsson et al., 2010). Since these patterns were not observed, it is possible to consider that the dispersal process does not modulate the diversity patterns of riparian plant communities in this mountain river. Future studies should recognize whether this pattern is consistent in other mountain rivers since our study was carried out only in a river.

The increase in dissimilarity in composition with geographic distance that was observed in the decay‐similarity analysis has been extensively studied in ecological studies and has been observed in plant communities in riparian zones (Kuglerova et al., 2015; Warfe et al., 2013) as well as for other organisms and environments (Nekola & White, 1999). Two causes have been proposed to explain distance decay: (a) decrease in environmental similarity with distance and (b) landscape restrictions related to the dispersal capacities of organisms (Soininen et al., 2007). This last explanation was not possible to analyze in this work, since we did not record measures related to the limitation of the movement of organisms; however, we do find support to suggest that changes in environmental conditions may be behind this pattern in riparian plant communities.

In this study, environmental dissimilarity increased with the increase in the distance to the origin of the river, that is, there are strong environmental gradients along the river that may be modulating the changes in species richness and composition of the riparian plant communities. This is concordant with studies that have observed that propagule dispersal does not structure the riparian plant communities and that it suggested that changes in species richness and composition of the riparian communities are related to environmental changes along rivers (Jiang et al., 2005; Tab acchi et al., 1990; Warfe et al., 2013). In rivers associated with mountain systems, wide environmental gradients are presented due to the drastic altitude changes, the associated climatic variation, and the geomorphology that characterize mountain rivers (Hupp & Osterkamp, 1996).

The importance of environmental gradient in changes in species richness and in the composition of communities is also supported by the results of the analysis of the relationship with environmental variables. Altitude was one of the most important variables to explain the changes observed in species richness and in the composition of communities along the river. The importance of altitude in changes in the diversity of riparian plant communities in mountain rivers has been observed to be the result of the association between altitude with changes in climatic conditions (Rico et al., 2006; Salinas & Casas, 2007); these variations could determine the presence of species associated with certain ranges of temperature and precipitation (Jiang et al., 2005). Such is the case of *Abies hickelli*, which is found in areas with a temperature of 11.1–13.7°C and an average annual rainfall of 1,332–1,591 mm (Gutiérrez & Trejo, 2014), these conditions are only found in the highest of the study area and that coincide with the presence of this species at sites above 2,800 m a.s.l. (sites 1, 2, and 3).

Altitude is also associated with changes in soil variables such as pH and conductivity of riparian zones (Sagers & Lyon, 1997; Salinas & Casas, 2007). In this work, we observe a relationship between the altitude and the edaphic variables of conductivity and temperature. Conductivity was one of the variables that were related to the decrease in species richness and changes in composition in the upper and the middle strata along the river. The relationship of electrical conductivity to species richness and composition is likely due to the accumulation of ions in the soil from areas that are frequently flooded (Ou et al., 2019). The accumulation of ions such as Fe^2+^ and Mg^2+^ can have a negative effect on species richness and cause changes in the composition of species in the riparian zone by limiting the absorption of nutrients in plants (Colmer & Voesenek, 2009). Toward downstream, the frequency and duration of floods are greater compared with sites at the headwaters of the basin, since the volume of water transported by rivers increases downstream (Wohl, 2010). These changes in edaphic conditions downstream are an environmental filter that limits the establishment of plants that cannot maintain their functions under flood conditions (Colmer & Voesenek, 2009; Wohl, 2010). This is one of the reasons why toward the areas furthest from the origin of the river, the importance of riparian species such as *Alnus acuminata* and *Salix bonplandiana* was greater, while species that can develop below 2,000 m a. s. l. like *Oreopanax xalapensis* were not observed due to lack of adaptations to survive in flood conditions (Pennington, 2005).

Finally, the river flow was another of the variables of great importance that helped explain the changes in the species richness and composition of the communities along the river. This may be the result of the disturbance effect generated by fluvial dynamics in rivers with large channels (Bendix & Hupp, 2000; Steiger et al., 2005). A river with a higher flow transport more water, this greater amount of water generate greater disturbance in riparian communities further away from the river origin site because they extend the periods of flooding and a greater erosion of the margins of the rivers due to the greater kinetic energy (Bendix & Hupp, 2000; Steiger et al., 2005; Wohl, 2010).

Although our results show that changes in the environmental variables (topographic, hydrologic, and edaphic) along the river are related to changes in the diversity of riparian plant communities that develop in mountain rivers, we could not rule out a possible effect of propagule dispersal, since we do not include propagule dispersal as an explanatory variable. We establish two possible hypotheses by which the propagule dispersal process did not determine the patterns of diversity, according to the theoretical framework of the HRC and our hypotheses: (a) along a mountain river there is no greater deposition of propagules in the riparian zones with the increase in the distance to the site of origin of the river, because the erosive capacity exceeds that of deposition, so that the propagules that are transported in the body of water are not deposited in the riparian zone, on the other hand, if they are deposited they are quickly removed by the water flow to downstream riparian zones that develop outside the mountain area and (b) the propagules are effectively transported and deposited in the riparian zones, but environmental filters do not allow their germination or establishment because they do not have the mechanisms to stay in place.

Another aspect to consider is the environmental changes throughout the year since differences were observed in the edaphic variables of conductivity and moisture. These are related to changes in precipitation between the dry and the rainy season, which modify the water level in rivers and with it the conductivity and moisture in the riparian zone (Ou et al., 2019; Stromberg et al., 2010). Despite the differences between seasons, we observed that the conductivity and moisture had a similar behavior along the river in the dry and rainy seasons. This does not imply that these temporal changes do not have an effect on communities; several studies have found that seasonal changes are important for riparian communities (Bendix & Hupp, 2000; Greet et al., 2011; Stromberg et al., 2010). It is likely that, notably in the lower stratum, different species are present in the different seasons. However, changes in plant communities between seasons were not considered in this work. Future work could study the changes in communities between seasons along the river and evaluate: (a) changes in diversity patterns between seasons and (b) if changes in environmental variables between seasons are related to possible changes in the patterns of diversity of riparian plant communities along mountain rivers.

A final consideration must be made regarding the collinearity between the environmental variables (especially the edaphic ones) and the confounding effect that it may have generated in the variables that were found to be most important for the changes we observed in the riparian communities. Three key problems arise under collinearity: (a) reverse interpretation (the estimated coefficients have the wrong sign), (b) suppression or masking (the apparent absence of relationship between the predictor and the response is false or this decreased), and (c) dangerous extrapolation (the prediction is restricted to the sample space, the extrapolation beyond the data is dangerous) (Dormann et al., 2013; Meloun et al., 2002). We did not find evidence of inverse interpretation, since no changes were observed in the sign of the estimated coefficients in the LASSO path (Appendix S3); however, we have evidence of suppression in the model for the upper stratum since the LASSO regression model consider two more variables (flow of the river and moisture content of the soil) that the GLMs exclude. Considering that this study was carried out in a single river, it is possible to expect dangerous extrapolation as well (although predicting the magnitude of change was not an objective of the study). A possible solution to these last two problems is to increase the sampling effort to other mountain rivers and observe whether the environmental variables that we found to be important for the changes in species richness are also important in other areas.

## 5 CONCLUSION

Our study shows that it is possible to observe changes in the species richness and composition of riparian plant communities in mountain rivers that differ from those patterns that have been observed in rivers with small altitude gradients on large spatial scales. These changes respond significantly to changes in environmental conditions that occur along the river and that are associated with soil variables, hydrological dynamics, and altitudinal contrast. Considering these results, it is necessary to review the applicability of the "River Collector Hypothesis" in riparian mountain systems and propose alternative mechanisms that help us explain the diversity patterns of riparian plant communities that develop in mountain systems.

## CONFLICT OF INTEREST

The authors declare that we have no conflict of interest.

## AUTHOR CONTRIBUTION

**Nihaib Flores:** Conceptualization (lead); Data curation (lead); Formal analysis (lead); Methodology (equal); Validation (lead); Writing‐original draft (lead); Writing‐review & editing (lead). **Trejo Irma:** Funding acquisition (lead); Methodology (equal); Resources (lead); Supervision (equal); Writing‐review & editing (lead). **Neptalí Ramírez‐Marcial:** Methodology (equal); Supervision (equal); Writing‐review & editing (supporting).

## Supporting information

Supplementary Material

## Data Availability

Sample location and vegetation sampling are available on Dryad https://doi.org/10.5061/dryad.sf7m0cg5r.
