# Supplementary material for: Environment‐driven changes in diversity of riparian plant communities along a mountain river
Source: Ecol Evol. 2021 Mar 25;11(10):5690–701. doi: 10.1002/ece3.7472 (PMC8131774; doi:10.1002/ece3.7472)
Supplement: Supplementary file 1 — Supplementary Material [file ECE3-11-5690-s001.docx]

**Supporting information to the paper**

Flores-Galicia, N et al. 2021. Environment driven changes in riparian plant communities along a mountain river. *Ecology and Evolution*.

**Appendix S1**. Differences between seasons for soil variables. * Denotes significant statistical differences between seasons. * p=0.05, ** p=0.001, *** p=<0.0001.

| **Variable** | **Season** | | **Site** | | | | | |
| --- | --- | --- | --- | --- | --- | --- | --- | --- |
|  |  |  | 1 | 2 | 3 | 10 | 11 | 14 |
| Conductivity  (µS/cm)** | Wet | $\bar{x}$ | 103.33 | 60 | 69.44 | 124.44 | 138.89 | 97.78 |
|  |  | D.E. | 39.85 | 44.05 | 40.51 | 63.45 | 105.99 | 110.32 |
|  | Dry | $\bar{x}$ | 56.67 | 38.33 | 50 | 96.67 | 155 | 93.33 |
|  |  | D.E. | 45.02 | 32.51 | 38.99 | 111.83 | 100.15 | 70.90 |
| Moisture  (%)** | Wet | $\bar{x}$ | 9.69 | 7.42 | 9.04 | 12.49 | 12.73 | 7.92 |
|  |  | D.E. | 3.09 | 2.74 | 2.16 | 3.10 | 4.71 | 6.04 |
|  | Dry | $\bar{x}$ | 8.07 | 6.48 | 6.20 | 9.07 | 9.62 | 7.97 |
|  |  | D.E. | 2.43 | 3.39 | 4.50 | 6.97 | 4.09 | 6.48 |
| pH | Wet | $\bar{x}$ | 6.38 | 6.46 | 6.46 | 6.14 | 6.01 | 6.54 |
|  |  | D.E. | 0.52 | 0.37 | 0.42 | 0.45 | 0.51 | 0.65 |
|  | Dry | $\bar{x}$ | 6.78 | 6.62 | 6.72 | 6.67 | 6.18 | 6.43 |
|  |  | D.E. | 0.24 | 0.39 | 0.31 | 0.41 | 0.46 | 0.62 |
| Temperature  (°C)*** | Cold | $\bar{x}$ | 7.01 | 7.71 | 7.73 | 10.23 | 11.07 | 10.02 |
|  |  | D.E. | 2.54 | 2.42 | 2.46 | 1.71 | 0.78 | 0.59 |
|  | Heat | $\bar{x}$ | 10.05 | 10.72 | 10.84 | 14.17 | 14.11 | 14.56 |
|  |  | D.E. | 0.67 | 0.51 | 0.35 | 0.69 | 0.65 | 0.56 |

**Appendix S2**. Correlation (*r* of Pearson) between environmental variables. Cha=channel width, Con=conductivity, Mc=moisture content, Tem=temperature, Flo=flow, Alt=altitude, Dis=distance to the origin of river. The edaphic variables in which differences were observed between seasons are shown (Appendix S1). (We)=wet, (Dr)= dry, (Co)=cold, (He)=heat.

|  | Con (We) | Con (Dr) | Mc (We) | Mc (Dr) | Ph | Tem (Co) | Tem (He) | Cha | Flo | Alt | Dis |
| --- | --- | --- | --- | --- | --- | --- | --- | --- | --- | --- | --- |
| Con  (We) | - |  |  |  |  |  |  |  |  |  |  |
| Con  (Dr) | 0.89 | - |  |  |  |  |  |  |  |  |  |
| Mc  (We) | 0.87 | 0.73 | - |  |  |  |  |  |  |  |  |
| Mc  (Dr) | 0.98 | 0.86 | 0.79 | - |  |  |  |  |  |  |  |
| Ph | -0.83 | -0.88 | -0.87 | -0.8 | - |  |  |  |  |  |  |
| Tem  (Co) | 0.72 | 0.9 | 0.56 | 0.73 | -0.73 | - |  |  |  |  |  |
| Tem  (He) | 0.66 | 0.81 | 0.45 | 0.68 | -0.57 | 0.97 | - |  |  |  |  |
| Cha | 0.5 | 0.71 | 0.39 | 0.48 | -0.48 | 0.92 | 0.95 | - |  |  |  |
| Flo | 0.46 | 0.73 | 0.16 | 0.47 | -0.37 | 0.87 | 0.91 | 0.9 | - |  |  |
| Alt | -0.6 | -0.79 | -0.37 | -0.62 | 0.52 | -0.96 | -0.99 | -0.96 | -0.95 | - |  |
| Dis | 0.62 | 0.78 | 0.36 | 0.63 | -0.47 | 0.94 | 0.98 | 0.95 | 0.96 | -0.99 | - |

**Appendix S3**. Lasso path for the changes in species richness in the three strata (Upper, middle and low) and environmental variables. Alt=altitude, Con=conductivity, Flo=flow, Mc=moisture content. It is observed that with the decrease in the penalty value (lambda) the number of variables that are added to the model increases. Furthermore, no sign changes are detected in the relationship between a variable and species richness when new variables are added to the model.


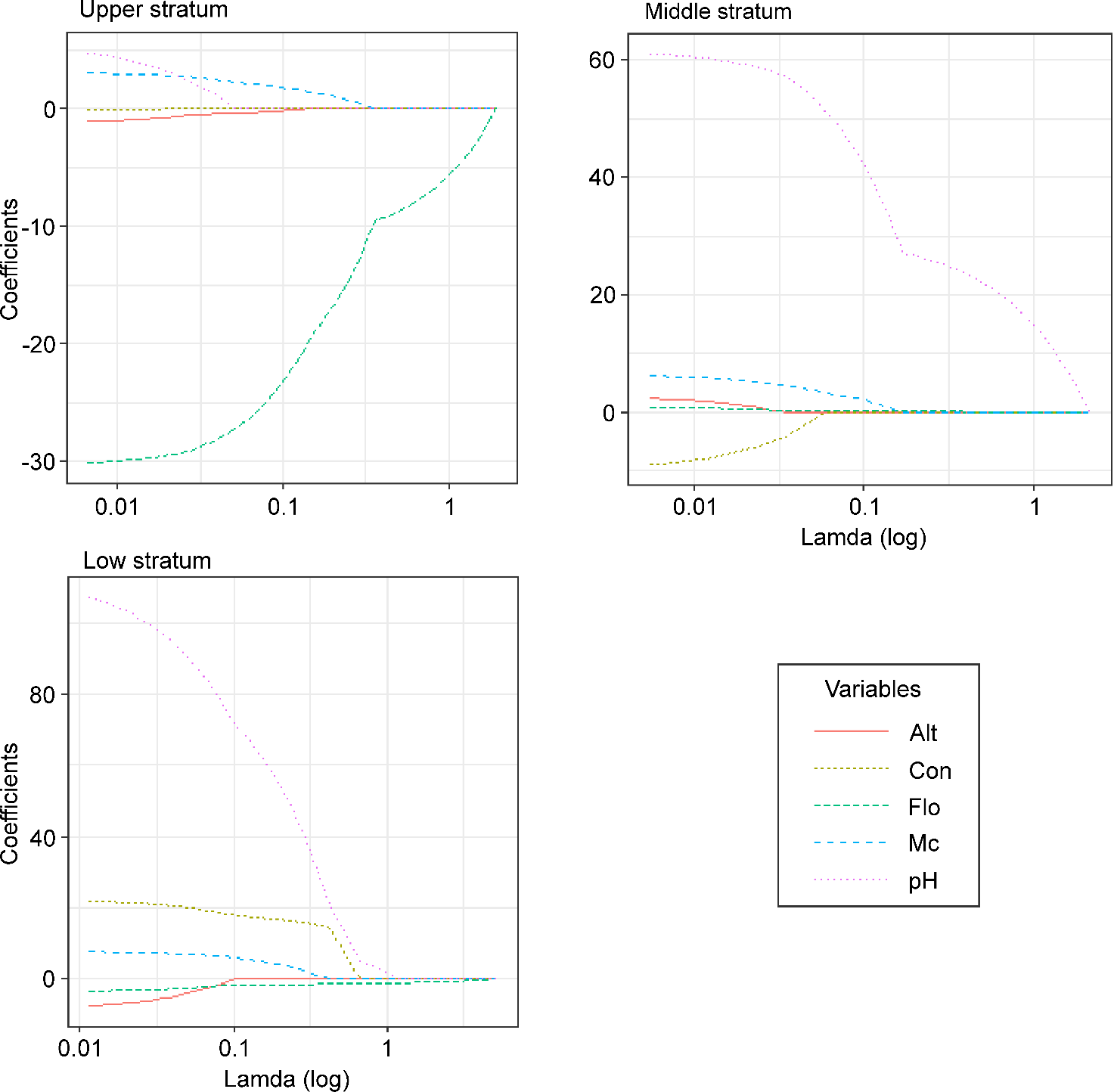


**Appendix S4**. The species relative importance value (RIV) in each site for the upperestratum (A), middles tratum (B) and for the low stratum (C).

| Upper stratum | | | | | | | | | | | | | | | |
| --- | --- | --- | --- | --- | --- | --- | --- | --- | --- | --- | --- | --- | --- | --- | --- |
| Species/key | Site | | | | | | | | | | | | | | |
|  | 1 | 2 | 3 | 4 | 5 | 6 | 7 | 8 | 9 | 10 | 11 | 12 | 13 | 14 | 15 |
| *Abies hickelli*/Abi hick | 33 | - | 3 | - | - | - | - | - | - | - | - | - | - | - | - |
| *Ageratina pazcuarensis*/Age pazc | 11 | - | 3 | - | - | - | - | - | - | - | 3 | - | - | - | - |
| *Ageratina petiolaris*/Age peti | 3 | - | - | - | - | - | - | - | - | - | - | - | 6 | - | - |
| *Ageratin*a sp. 2/Age 2 | - | - | - | - | - | - | - | - | - | - | - | 8 | - | - | - |
| *Alloispermum integrifolium*/All inte | - | - | - | - | - | - | - | - | - | - | - | - | - | 1 | - |
| *Alnus acuminata*/Aln acum | - | - | - | - | 44 | 28 | 40 | 45 | 33 | 14 | 34 | 30 | 42 | 45 | 54 |
| *Annona cherimola*/Ann cher | - | - | - | - | - | - | - | - | - | - | - | - | - | 2 | 7 |
| *Arracacia aegopodioides*/Arr aego | - | 1 | - | - | - | - | - | - | - | - | - | - | - | - | - |
| Asteraceae sp. 6/Ast 6 | - | - | - | - | - | - | - | - | - | - | - | - | 3 | - | 2 |
| *Baccharis conferta*/Bac conf | - | - | - | - | - | 5 | - | - | 2 | - | - | 5 | 8 | 9 | 3 |
| *Baccharis salicifolia*/Bac sali | - | - | - | - | - | - | 3 | - | - | - | - | - | 5 | 9 | - |
| *Berberis moranensis*/Ber mora | - | - | 1 | - | - | - | - | - | - | - | - | - | - | - | - |
| *Brickellia orizabensis*/Bri oriz | - | - | - | - | - | - | - | - | - | - | 9 | - | - | 2 | - |
| *Buddleja cordata*/Bud cord | - | - | - | - | 2 | - | 3 | - | - | - | - | - | - | - | - |
| *Buddleja parviflora*/Bud parv | - | - | - | - | - | - | - | - | 2 | - | - | - | - | - | - |
| *Calliandra houstoniana*/Cal hous | - | - | - | - | - | - | - | - | 4 | - | - | - | - | - | - |
| *Ceanothus caeruleus*/Cea caer | - | 2 | - | - | - | - | - | - | - | - | - | - | - | - | - |
| *Cedrela oaxacensis*/Ced oaxa | - | - | - | - | - | - | - | - | - | - | 2 | 9 | - | - | 2 |
| *Cestrum laxum*/Ces laxu | 2 | - | 5 | - | 13 | 11 | 8 | 13 | - | 2 | 5 | 2 | - | - | - |
| *Cestrum roseum*/Ces rose | - | - | - | - | - | - | - | - | - | - | 3 | - | - | - | - |
| *Clematis dioica*/Cle dioi | - | - | - | - | - | - | - | - | - | - | - | 2 | 2 | 1 | - |
| *Clethra gaelottiana*/Cle gael | - | 13 | 4 | 23 | 6 | - | - | - | - | - | - | - | - | - | - |
| *Clinopodium macrostemum*/Cli macr | - | - | - | 1 | - | - | - | - | - | - | - | - | - | - | - |
| *Comarostaphylis discolor*/Com disc | 6 | 6 | - | - | - | - | - | - | - | - | - | - | - | - | - |
| *Cornus disciflora*/Cor disc | - | - | 4 | 2 | 2 | 3 | - | - | - | - | - | - | - | - | - |
| *Cornus excelsa*/Cor exce | - | - | - | - | - | - | - | - | - | 11 | - | - | - | - | - |
| *Equisetum hyemale*/Equ hyem | - | - | - | - | - | - | - | - | - | - | - | - | - | 2 | - |
| *Fraxinus uhdei*/Fra uhde | - | - | - | - | 18 | 7 | 7 | 4 | - | - | - | - | - | - | - |
| *Fuchsia microphylla*/Fuc micr | - | - | - | - | - | - | - | - | 2 | - | - | - | - | - | - |
| *Garrya laurifolia*/Gar laur | 7 | 5 | 4 | 8 | - | - | - | - | - | - | - | - | - | - | - |
| *Iresine diffusa*/Ire diff | - | - | - | - | - | - | - | - | - | 2 | - | - | - | - | - |
| Litsea glaucescens/Lit glau | - | 10 | 4 | 4 | - | - | - | 7 | - | - | - | - | - | - | - |
| *Lonicera mexicana*/Lon mexi | 2 | 3 | - | - | - | - | - | - | - | - | - | - | - | - | - |
| *Meliosma dentata*/Mel dent | - | 22 | 23 | 7 | - | 3 | 9 | 2 | - | 6 | - | - | - | - | - |
| *Monnina ciliolata*/Mon cili | 2 | 1 | - | - | - | - | 3 | - | 2 | 4 | - | - | - | - | - |
| *Montanoa karwinskii*/Mon karw | - | - | - | - | - | - | - | - | - | - | - | - | - | - | 10 |
| *Montanoa leucantha* subsp. *arborescen*s/Mon arbo | - | - | - | - | - | - | - | - | - | - | - | - | - | 1 | - |
| *Myrsine juergensenii*/Myr juer | - | - | - | - | - | 8 | 4 | 4 | 11 | 22 | 2 | - | - | - | - |
| *Oreopanax xalapensis*/Ore xala | - | 5 | 3 | - | 6 | 2 | 6 | 4 | 7 | 19 | 2 | - | - | - | - |
| *Ostrya virginiana*/Ost virg | - | - | - | - | - | - | - | 2 | - | - | - | - | - | - | - |
| *Persea americana*/Per amer | - | - | - | - | - | - | - | - | - | - | - | 4 | - | - | - |
| *Philadelphus mexicanus*/Phi mexi | - | - | - | - | - | - | - | 2 | - | - | - | 10 | - | - | - |
| *Pinus* sp./Pin sp. | - | 2 | - | 11 | - | 6 | - | - | - | - | - | - | - | - | - |
| *Prunus brachybotrya*/Pru brac | - | - | 6 | - | 2 | 16 | 10 | 10 | 8 | 20 | 9 | 20 | 2 | - | - |
| *Prunus serotina* subsp. *capul*i/Pru capu | - | - | - | - | - | - | - | - | - | - | - | 4 | 3 | - | - |
| Quercu*s acutifolia*/Que acut | - | - | - | - | 4 | - | - | - | - | - | 2 | - | - | - | - |
| *Quercus glabrescens*/Que glab | - | 9 | - | 3 | - | - | - | - | - | - | - | - | - | - | - |
| *Quercus laurina*/Que laur | - | - | - | 3 | - | - | - | - | - | - | - | - | - | - | - |
| *Quercus obtusifolia*/Que obtu | - | - | - | - | - | 10 | - | - | - | - | - | - | - | - | - |
| *Querc*us sp. 5/Que 5 | - | - | - | - | - | - | - | - | 4 | 2 | - | - | - | - | - |
| *Rhynchosia minima*/Rhy mini | - | - | - | - | - | - | - | - | - | - | - | - | - | 2 | - |
| *Robinsonella cordata*/Rob cord | - | - | - | - | - | - | - | - | - | - | - | - | - | 1 | - |
| *Roldana barba-johannis*/Rol barb | - | 7 | 7 | 7 | - | - | - | - | - | - | - | - | - | - | - |
| *Roldana jurgensenii*/Rol jurg | 25 | 1 | 2 | 3 | - | - | 6 | - | 4 | - | - | - | 2 | - | - |
| *Rubus adenotrichos*/Rub aden | - | - | - | - | - | - | - | - | - | - | 2 | - | - | - | - |
| *Rubus pringlei*/Rub prin | 2 | - | - | - | - | - | - | - | - | - | - | - | - | - | - |
| *Salix bonplandiana*/Sal bonp | - | - | - | - | - | - | - | 2 | 7 | - | 21 | 5 | 20 | 23 | 22 |
| *Salix paradoxa*/Sal para | 5 | - | - | - | - | - | - | - | - | - | - | - | - | - | - |
| *Sauraria serrata*/Sau serr | - | - | 1 | - | - | - | - | - | - | - | - | - | - | - | - |
| *Senecio callosus*/Sen call | 2 | - | - | - | - | - | - | - | - | - | - | - | - | - | - |
| *Serjania schiedeana*/Ser schi | - | - | - | - | - | - | - | - | - | - | 2 | - | - | - | - |
| *Solanum cervantesii*/Sol cerv | - | - | - | - | - | - | 3 | 2 | - | - | - | - | - | - | - |
| *Solanum rostratum*/Sol rost | - | - | - | - | - | - | - | - | - | - | - | - | 6 | - | - |
| Sp. 11/Sp. 11 | - | - | - | 1 | - | - | - | - | - | - | - | - | - | - | - |
| Sp. 24/Sp. 24 | - | - | - | 1 | - | - | - | - | - | - | - | - | - | - | - |
| *Telanthophora andrieuxii*/Tel andr | - | 12 | 30 | 22 | 2 | - | - | - | - | - | - | - | - | - | - |
| *Tilia* americana/Til amer | - | - | - | - | - | - | - | - | 14 | - | - | - | - | - | - |
| *Verbesina hypoglauca*/Ver hypo | - | - | - | 2 | - | - | - | - | - | - | - | - | - | - | - |
| *Viburnum elatum*/Vib elat | - | - | 1 | 1 | - | - | - | - | - | - | - | - | - | - | - |
| *Viburnum stenocalyx*/Vib sten | - | - | - | - | - | - | - | 3 | - | - | - | - | - | - | - |
| *Vitis tiliifolia*/Vit tili | - | - | - | - | - | - | - | - | - | - | 2 | - | 2 | - | - |

| (B) Middle stratum | | | | | | | | | | | | | | | |
| --- | --- | --- | --- | --- | --- | --- | --- | --- | --- | --- | --- | --- | --- | --- | --- |
| Specise/key | Site | | | | | | | | | | | | | | |
|  | 1 | 2 | 3 | 4 | 5 | 6 | 7 | 8 | 9 | 10 | 11 | 12 | 13 | 14 | 15 |
| *Abies hickelli*/Abi hick | 5 | 19 | - | - | - | - | - | - | - | - | - | - | - | - | - |
| *Ageratina pazcuarensis*/Age pazc | 10 | - | - | - | - | - | - | - | - | - | - | - | - | - | - |
| *Ageratina petiolaris*/Age peti | - | - | - | - | - | - | - | - | - | - | - | - | 14 | - | - |
| *Alnus acuminata*/Aln acum | - | - | - | - | - | - | - | - | - | - | 22 | - | 24 | - | 26 |
| *Archibaccharis schiedeana*/Arc schi | - | - | - | - | 100 | - | - | - | - | - | - | - | - | - | - |
| *Baccharis conferta*/Bac conf | - | - | - | - | - | - | - | - | - | - | - | - | 26 | - | - |
| *Baccharis salicifolia*/Bac sali | - | - | - | - | - | - | - | - | - | - | - | - | - | 15 | - |
| *Brickellia orizabensis*/Bri oriz | - | - | - | - | - | - | - | - | - | - | 22 | - | - | 30 | - |
| *Calliandra houstoniana*/Cal hous | - | - | - | - | - | - | - | - | 13 | - | - | - | - | - | - |
| *Cestrum laxum*/Ces laxu | 5 | - | - | - | - | - | - | 25 | - | - | - | 19 | - | - | - |
| *Cestrum roseum*/Ces rose | - | - | - | - | - | - | - | - | - | - | 20 | - | - | - | - |
| *Clematis dioica*/Cle dioi | - | - | - | - | - | - | - | - | - | 7 | - | - | - | - | - |
| *Clinopodium macrostemum*/Cli macr | - | - | - | 12 | - | - | - | - | - | - | - | - | - | - | - |
| *Cornus excelsa*/Cor exce | - | - | - | - | - | - | - | - | - | 14 | - | - | 10 | - | - |
| *Equisetum hyemale*/Equ hyem | - | - | - | - | - | - | - | - | - | - | - | - | - | 43 | - |
| *Litsea glaucescens*/Lit glau | - | 12 | 11 | 22 | - | - | - | - | - | - | - | - | - | - | - |
| *Meliosma dentata*/Mel dent | - | 38 | - | - | - | - | - | - | - | - | - | - | - | - | - |
| *Monnina ciliolata*/Mon cili | - | - | - | - | - | - | 17 | 26 | 13 | 15 | - | - | - | - | - |
| *Myrsine juergensenii*/Myr juer | - | - | - | - | - | - | - | - | - | - | 22 | - | - | - | - |
| *Oreopanax xalapensis*/Ore xala | - | - | 11 | 6 | - | 14 | 22 | - | 22 | - | - | - | - | - | - |
| *Philadelphus mexicanus*/Phi mexi | - | - | - | 4 | - | - | - | - | - | - | - | - | - | - | - |
| *Pinus* sp./Pin sp. | - | - | - | - | - | - | - | - | - | 9 | - | - | - | - | - |
| *Prunus brachybotrya/*Pru brac | - | - | - | - | - | 33 | - | 10 | 13 | 29 | - | 62 | - | - | - |
| *Quercus acutifolia*/Que acut | - | - | - | - | - | - | - | 15 | - | - | - | - | - | - | - |
| *Quercus glabrescens*/Que glab | - | - | - | 6 | - | - | - | - | - | - | - | - | - | - | - |
| *Roldana barba-johannis*/Rol barb | - | 19 | 23 | - | - | - | - | - | - | - | - | - | - | - | - |
| *Roldana jurgensenii*/Rol jurg | 64 | - | 15 | 6 | - | - | - | - | 11 | - | - | - | - | - | - |
| *Rubus adenotrichos*/Rub aden | - | - | - | - | - | - | - | - | - | - | 15 | - | - | - | - |
| *Rubus pringlei*/Rub prin | 6 | - | - | - | - | - | - | - | - | - | - | - | - | - | - |
| *Salix bonplandiana*/Sal bonp | - | - | - | - | - | - | - | - | - | - | - | - | 15 | 12 | 74 |
| *Salvia atropaenulata*/Sal atro | - | - | - | - | - | 37 | 32 | - | - | 17 | - | - | - | - | - |
| *Scutellaria dumetorum*/Scu dume | - | 13 | - | - | - | - | - | - | - | - | - | - | - | - | - |
| *Senecio callosus*/Sen call | 4 | - | - | - | - | - | - | - | - | - | - | - | - | - | - |
| *Smilax moranensis*/Smi mora | 5 | - | - | 5 | - | - | - | - | - | - | - | - | - | - | - |
| *Solanum cervantesii*/Sol cerv | - | - | - | - | - | - | 29 | 24 | - | - | - | - | - | - | - |
| *Solanum nigricans*/Sol nigr | - | - | - | - | - | 16 | - | - | - | - | - | - | - | - | - |
| Sp. 23/Sp. 23 | - | - | 28 | - | - | - | - | - | - | - | - | - | - | - | - |
| *Telanthophora andrieuxii*/Tel andr | - | - | 11 | 6 | - | - | - | - | - | - | - | - | - | - | - |
| *Tilia americana*/Til amer | - | - | - | - | - | - | - | - | 13 | - | - | - | - | - | - |
| *Viburnum elatum*/Vib elat | - | - | - | 6 | - | - | - | - | - | - | - | - | - | - | - |
| *Vitis tiliifolia*/Vit tili | - | - | - | - | - | - | - | - | - | - | - | - | 10 | - | - |
| *Valeriana naidae*/Val naid | - | - | - | - | - | - | - | - | - | - | - | 19 | - | - | - |
| *Rhamnus mucronata*/Rha mucr | - | - | - | - | - | - | - | - | 13 | - | - | - | - | - | - |
| *Tournefortia hirsutissima*/Tou hirs | - | - | - | - | - | - | - | - | - | 8 | - | - | - | - | - |

| (C) Low stratum | | | | | | | | | | | | | | | |
| --- | --- | --- | --- | --- | --- | --- | --- | --- | --- | --- | --- | --- | --- | --- | --- |
| Species/key | Site | | | | | | | | | | | | | | |
|  | 1 | 2 | 3 | 4 | 5 | 6 | 7 | 8 | 9 | 10 | 11 | 12 | 13 | 14 | 15 |
| *Abies hickelii*/Abi hick | - | 5 | - | - | - | - | - | - | - | - | - | - | - | - | - |
| *Adiantum* sp./Adi sp. | - | - | - | - | - | - | - | - | 12 | - | 8 | - | - | - | - |
| *Ageratina pazcuarensis*/Age pazc | 4 | - | - | - | - | - | - | - | - | - | - | - | - | - | - |
| *Ageratina petiolaris*/Age peti | - | - | - | - | - | - | - | - | - | - | - | 9 | 11 | - | - |
| *Ageratina* sp. 2/Age 2 | - | - | - | - | 7 | - | - | - | - | - | - | - | - | - | - |
| *Alchemilla procumbens*/Alc proc | - | - | - | 17 | - | - | - | - | - | - | - | - | - | - | - |
| *Alchemilla* sp. 1/Alc 1 | 3 | - | - | - | - | - | - | - | - | - | - | - | - | - | - |
| *Alchemilla vulcanica*/Alc vulc | - | - | - | 4 | - | - | - | - | - | - | - | - | - | - | - |
| *Alnus acuminata*/Aln acum | - | - | - | - | - | - | - | - | - | 6 | - | - | - | - | - |
| Apocynaceae sp. 2/Apo 2 | - | - | - | - | - | - | - | 14 | - | - | - | - | - | - | - |
| *Archibaccharis auriculata*/Arc auri | 4 | - | - | - | - | - | - | - | - | - | - | - | - | - | - |
| *Arracacia aegopodioides*/Arr Arra | - | - | 4 | - | - | - | - | - | - | - | - | - | - | - | - |
| *Asplenium* sp. 2/Asp 2 | - | 4 | - | 4 | - | - | - | - | - | - | - | - | - | - | - |
| *Asplenium* sp. 3/Asp 3 | - | - | - | - | 8 | - | - | - | - | - | - | - | - | - | - |
| Asteraceae sp. 14/Ast 14 | - | - | - | - | - | - | - | - | - | 5 | - | - | - | - | - |
| Asteraceae sp. 37/Ast 37 | - | - | - | - | - | - | - | - | - | - | - | - | - | 14 | - |
| Asteraceae sp. 39/Ast 39 | - | - | - | - | - | - | - | - | - | - | - | - | - | 9 | - |
| Asteraceae sp. 46/Ast 46 | 3 | - | - | - | - | - | - | - | - | - | - | - | - | - | - |
| Asteraceae sp. 5/Ast 5 | - | - | - | - | - | - | - | - | 20 | - | - | - | - | - | - |
| Asteraceae sp. 75/Ast 75 | - | - | - | - | - | - | - | - | - | - | - | - | - | - | 4 |
| *Baccharis conferta*/Bac conf | - | - | - | - | - | - | - | - | - | - | - | - | - | 11 | - |
| *Bomarea acutifolia*/Bom acut | - | - | 4 | - | - | - | - | - | - | - | - | - | - | - | - |
| *Calliandrahoustoniana*/Cal Call | - | - | - | - | - | - | - | - | 12 | - | - | - | - | - | - |
| *Cardamine fulcrata*/Car fulc | - | - | - | - | - | - | 25 | - | - | 4 | - | - | - | - | - |
| *Cestrum laxum*/Ces laxu | - | - | - | - | - | - | - | - | - | - | 5 | - | - | - | - |
| *Cheilanthes* sp. 2/Che 2 | - | 5 | - | - | - | - | - | - | - | - | - | - | - | - | - |
| *Cleome magnifica*/Cle magn | - | - | - | - | - | - | - | - | - | 9 | - | - | - | - | - |
| *Clethra gaelottiana*/Cle gael | - | 5 | - | - | - | - | - | - | - | - | - | - | - | - | - |
| *Crocosmia x crocosmiiflora*/Cro croc | - | - | - | - | - | - | - | - | - | - | - | - | 24 | - | - |
| *Crusea coccinea*/Cru cocc | 5 | 40 | 49 | 24 | 20 | - | - | - | - | - | - | - | - | - | - |
| *Cuphea cyanea*/Cup cyan | - | - | - | - | - | - | 11 | - | - | - | - | - | - | - | - |
| *Cyperus* sp. 1/Cyp 1 | - | - | - | - | - | - | - | - | 20 | - | - | - | - | - | - |
| *Danaea* sp./Dan sp. | - | - | - | 6 | - | - | - | - | - | - | - | - | - | - | - |
| *Dicliptera unguiculata*/Dic ungu | - | - | - | - | - | - | - | - | - | - | - | - | 40 | 16 | - |
| *Didymaea floribunda*/Did flor | 4 | - | 5 | - | - | - | - | - | - | - | - | - | - | - | - |
| *Drymaria villosa*/Dry vill | - | 9 | - | - | - | - | - | - | - | 5 | - | - | - | - | - |
| *Dysphania ambrosoides*/Dys ambr | - | - | - | - | - | - | - | - | - | - | - | - | - | - | 19 |
| *Echeveria rosea*/Ech rose | - | - | - | - | - | 11 | - | - | - | - | - | - | - | - | - |
| *Equisetum hyemale*/Equ hyem | - | - | - | - | - | - | - | - | - | - | - | - | - | - | 7 |
| *Eryngium* sp./Ery sp. | - | - | - | - | - | - | - | - | - | - | - | - | 7 | - | - |
| *Eryngium deppeanum*/Ery Eryn | 2 | - | - | - | - | - | - | - | - | - | - | - | - | - | - |
| *Fuchsia microphylla*/Fuc micr | - | - | - | - | - | - | - | - | - | - | 5 | - | - | - | - |
| *Geranium schiedeanum*/Ger schi | 6 | - | - | - | - | - | - | - | - | - | - | - | - | - | - |
| *Gibasis pellucida*/Gib Giba | - | - | - | - | - | - | - | - | - | - | - | 80 | - | - | - |
| *Gonolobus nemorosus*/Gon nemo | - | - | - | - | - | - | - | - | 16 | - | 11 | - | - | - | - |
| *Heuchera orizabensis*/Heu oriz | 3 | 5 | - | - | 9 | - | - | - | - | - | - | - | - | - | - |
| *Ipomoea* sp./Ipo sp. | - | - | - | - | - | - | - | - | - | 7 | - | - | - | - | - |
| *Iresine diffusa*/Ire diff | - | - | - | - | 16 | - | 11 | - | - | - | - | - | - | - | - |
| Iridaceae sp. 1/Iri 1 | - | - | - | - | - | - | - | - | - | 8 | - | - | - | - | - |
| Iridaceae sp. 2/Iri 2 | 3 | - | - | - | - | - | - | - | - | - | - | - | - | - | - |
| *Litsea glaucescens*/Lit glau | - | - | 10 | - | - | - | - | - | - | - | - | - | - | - | - |
| *Maianthemum comaltepecense*/Mai coma | - | 13 | 5 | - | - | - | - | - | - | - | - | - | - | - | - |
| *Monnina ciliolata*/Mon cili | - | - | - | - | - | - | - | - | - | 4 | - | - | - | - | - |
| *Myrsine juergensenii*/Myr juer | - | - | - | 4 | - | - | - | - | - | - | 11 | - | - | - | - |
| *Nertera granadensis*/Ner gran | 3 | 14 | 14 | 15 | - | - | - | - | - | - | - | - | - | - | - |
| *Oplismenus compositus*/Opl comp | - | - | - | - | - | - | - | - | - | - | - | - | - | 40 | - |
| *Oreopanax xalapensis*/Ore xala | - | - | 4 | - | - | 11 | - | - | - | - | - | - | - | - | - |
| *Orthrosanthus monadelphus*/Ort mona | - | - | - | - | - | - | - | - | - | - | - | - | 10 | - | - |
| *Osmorhiza mexicana*/Osm mexi | 4 | - | - | - | - | - | - | - | - | - | - | - | - | - | - |
| *Peperomia berlandieri*/Pep berl | - | - | - | 4 | - | 16 | - | - | - | - | - | - | - | - | - |
| *Peperomia dendrophila*/Pep dend | - | - | - | - | 8 | - | - | - | - | - | - | - | - | - | - |
| *Peperomia leptophylla*/Pep lept | - | - | - | - | - | - | 10 | - | - | 8 | - | - | 7 | - | - |
| *Persicaria capitata*/Per capi | - | - | - | - | - | - | - | - | - | - | - | - | - | - | 5 |
| *Pinus* sp./Pin sp. | - | - | - | 4 | - | - | - | - | - | - | - | - | - | - | - |
| Poaceae sp. 2/Poa 2 | 12 | - | - | - | - | - | - | - | - | - | - | - | - | - | - |
| Poaceae sp. 5/Poa 5 | - | - | - | - | - | - | - | - | - | - | - | - | - | - | 5 |
| Poaceae sp. 6/Poa 6 | - | - | - | - | - | - | - | - | - | - | - | - | - | - | 19 |
| *Polygonum laphatifolium*/Pol laph | - | - | - | - | - | - | - | - | - | - | - | - | - | - | 5 |
| *Polypogon elongatus*/Pol elon | - | - | - | - | - | - | - | - | - | 25 | - | - | - | - | - |
| *Prunus brachybotrya*/Pru brac | - | - | - | - | - | - | - | - | - | - | 16 | - | - | - | - |
| *Prunus serotina* subsp. *capuli*/Pru capu | - | - | - | - | - | - | - | - | - | - | 19 | - | - | - | - |
| *Pseudechinolaena* sp./Pse sp. | - | - | - | - | - | - | - | - | - | - | 6 | - | - | - | - |
| *Pteridium* sp. 1/Pte 1 | - | - | - | 6 | - | - | - | - | - | - | - | - | - | - | - |
| *Ranunculus* sp. 1/Ran 1 | 3 | - | - | - | - | - | - | - | - | - | - | - | - | - | - |
| *Roldana jurgensenii*/Rol jurg | 2 | - | - | 4 | - | - | - | - | - | - | - | - | - | - | - |
| *Rubus adenotrichos*/Rub aden | - | - | - | - | - | - | - | - | 20 | - | - | - | - | - | - |
| *Rubus pringlei*/Rub prin | 2 | - | - | - | - | - | - | - | - | - | - | - | - | - | - |
| *Rumex crispus*/Rum cris | - | - | - | - | - | - | - | - | - | - | - | - | - | - | 4 |
| *Salvia atropaenulata*/Sal atro | - | - | - | - | - | - | - | - | - | 6 | - | 20 | - | - | - |
| *Sedum oaxacanum*/Sed oaxa | 13 | - | - | - | - | - | - | - | - | - | - | - | - | - | - |
| *Senecio callosus*/Sen call | 6 | - | - | - | - | - | - | - | - | - | - | - | - | - | - |
| *Smilax moranensis*/Smi mora | - | - | 4 | 4 | - | - | - | 17 | - | - | - | - | - | - | - |
| *Solanum appendiculatum*/Sol appe | - | - | - | - | 33 | 62 | 43 | 68 | - | - | - | - | - | - | - |
| *Solanum cervantesii*/Sol cerv | - | - | - | - | - | - | - | - | - | - | 5 | - | - | - | 5 |
| *Solanum* sp./Sol sp. | - | - | - | - | - | - | - | - | - | - | - | - | - | - | 4 |
| Sp. 30/Sp. 30 | - | - | - | - | - | - | - | - | - | - | - | - | - | - | 7 |
| Sp. 31/Sp. 31 | - | - | - | - | - | - | - | - | - | - | - | - | - | - | 4 |
| Sp. 33/Sp. 33 | - | - | - | - | - | - | - | - | - | - | - | - | - | - | 4 |
| Sp. 5/Sp. 5 | - | - | - | - | - | - | - | - | - | 8 | - | - | - | - | - |
| Sp. 8/Sp. 8 | - | - | - | 4 | - | - | - | - | - | - | - | - | - | - | - |
| Sp. 9/Sp. 9 | 2 | - | - | - | - | - | - | - | - | - | - | - | - | - | - |
| *Stachys pilosissima*/Sta pilo | 4 | - | - | - | - | - | - | - | - | - | - | - | - | - | - |
| *Stellaria cuspidata*/Ste cusp | 3 | - | - | - | - | - | - | - | - | - | - | - | - | - | - |
| *Thelypteris* sp. 1/The 1 | - | - | - | - | - | - | - | - | - | - | 13 | - | - | - | - |
| *Tigridia orthantha*/Tig orth | 2 | - | - | - | - | - | - | - | - | - | - | - | - | - | - |
| *Veronica arvensis*/Ver arve | - | - | - | - | - | - | - | - | - | 6 | - | - | - | - | 4 |
| *Viola grahamii*/Vio grah | 9 | - | - | - | - | - | - | - | - | - | - | - | - | - | - |
| *Vitis tiliifolia*/Vit tili | - | - | - | - | - | - | - | - | - | - | - | - | - | - | 4 |
